# Ultrasonographic differentiation and Ultrasound-based management of partially cystic thyroid nodules

**DOI:** 10.20945/2359-3997000000367

**Published:** 2021-04-29

**Authors:** Yanjun Liu, Yanru Zhao, Jiao Fu, Shu Liu

**Affiliations:** 1 the First Affiliated Hospital of Xi'an Jiaotong University Department of Endocrinology Xi'an PR China Department of Endocrinology, the First Affiliated Hospital of Xi'an Jiaotong University, Xi'an, PR China

**Keywords:** Partially cystic thyroid nodules, ultrasonographic features, fine-needle aspiration biopsy

## Abstract

**Objective:**

To determine sonographic features of malignancy in partially cystic thyroid nodules and assess the diagnostic efficacy of these features for differentiating between benign and malignant lesions in the nodules with indeterminate cytology.

**Subjects and methods:**

From January 2016 to December 2017, a total of 91 patients with 94 partially cystic thyroid nodules who had undergone ultrasound-guided fine-needle aspiration biopsy and thyroid surgery in our hospital were included in this study. The sonographic features of the thyroid nodules were analyzed to identify the predictive features of malignancy and assess the diagnostic efficacy of these features.

**Results:**

The features of hypoechogenicity, microcalcification, composition, and an eccentric solid component with an acute angle had statistically significant associations with malignant nodule (*p*<005) by univariable analysis. Binary logistic regression analysis showed that microcalcification and hypoechogenicity were significantly associated with malignancy. Using the combination of microcalcification, hypoechogenicity, and a solid component comprising of greater than or equal to 50% of the total volume, the diagnostic sensitivity, specificity, positive predictive value (PPV), negative predictive value (NPV), and accuracy were 97.6%, 32.7%, 53.9%, and 94.4%, respectively. In these nodules with indeterminate cytology, this combination also exhibited a high sensitivity of 92.3% and an NPV of 83.3%.

**Conclusion:**

This study demonstrated that microcalcification and hypoechogenicity were independently associated with malignancy in partially cystic thyroid nodules. The combination of microcalcification, hypoechogenicity, and a solid portion that is greater than or equal to 50% of the total volume will help guide clinical decisions in mixed cystic solid nodules.

## INTRODUCTION

Thyroid nodules are a common clinical problem, occurring in 19-68% of the healthy population (1,2). Ultrasonography (US) is the primary imaging modality used to estimate the risk of malignancy in thyroid nodules. It also plays a crucial role in decisions regarding fine-needle aspiration (FNA) and a complementary role in the decisions involving medical management after an FNA is performed (3-6).

According to their composition, thyroid nodules can be classified as solid, mixed, or cystic based on a US evaluation. Partially cystic thyroid nodules (PCTNs) have both solid and cystic portions as a result of cystic degeneration of neoplastic or non-neoplastic nodules. They account for approximately 15% to 53.8% of all sonographically detected nodules (7-9). Most of them were considered benign lesions and hence managed conservatively. However, the percentage of malignancy in PCTNs varies from about 2% to 18% (7,8,10).

US features including hypoechogenicity, a taller-than-wide shape, the presence of microcalcifications, and lobulated/irregular margin are widely applied to differentiate benign from malignant nodules in solid thyroid nodules (6,11-14). However, sonographic features as predictors for risk of malignancy in PCTNs are different from that of solid nodules (7). Lee and cols. reported that a predominantly solid component, eccentric location, and microcalcification were associated with an increased risk of malignancy in PCTNs (8). In contrast, Na and cols. reported that only microcalcification was independently predictive of malignancy in PCTNs (7). In summary, US features predictive of malignancy in PCTNs have not been well established.

The purpose of the present study was to determine thyroid sonography features related to malignancy in PCTNs and assess the diagnostic efficacy of these features for differentiating between malignant and benign nodules with indeterminate cytology.

## SUBJECTS AND METHODS

### Patients

This retrospective study analyzed a total of 94 partially cystic thyroid nodules from 91 patients in the First Affiliated Hospital of Xi'an Jiaotong University between January 2016 to December 2017. Patients who met the following criteria were included in this study: (1) each nodule consisted of a solid and cystic component; (2) the patients underwent US-guided fine-needle aspiration biopsy (FNAB) based on the 2015 American Thyroid Association (ATA) guidelines or at the patients' request, and (3) the histopathologic result of each nodule was confirmed after surgery. US-guided FNAB was performed by a clinician with more than ten years of experience (>2,000 cases/year). Thyroid US records were reviewed by a radiologist (Yanjun, Liu) with four years of thyroid sonography experience (approximately 10,000 cases/year). The composition of the nodules based on US findings was classified as solid, mixed, or cystic. Purely cystic nodules indicated a completely anechoic nodule with or without a comet-tail artifact. PCTNs were characterized by the presence of both solid and cystic components. Only PCTNs where thyroid surgery was done were included in this study. The indications for thyroid surgery were a malignant or suspicious malignant cytology, or by the patients' choice. All patients signed informed consent concerning the future use of their clinical-pathological data for research purposes. This study was approved by the Ethics Committee of our hospital.

### Sonographic evaluation

Thyroid sonography was performed by a high-resolution sonographic instrument (NEMZ017, Toshiba, Japan) equipped with a 6- to 15-MHz linear probe. The sonographic images of PCTNs were retrospectively evaluated by an experienced radiologist blinded to the pathological results. The US features of all nodules were evaluated according to (1) composition, (2) percent of the solid component, (3) margin, (4) echogenicity of the solid portion, (5) presence of echogenic foci, (6) shape, and (7) halo.

The nodules were divided into the following three groups based on composition: group 1, a spongiform defined as the aggregation of multiple microcystic components in more than 50% of the nodule's volume; group 2, had a solid portion of less than 50% (not a pure cyst); and group 3, had a solid portion of greater than or equal to 50%.

According to the position of the solid portion, the nodules were classified as either eccentric or not. An eccentric nodule was defined as one with the solid portion not located in the center and abutted only on the side of the cyst wall. The eccentric configuration was subdivided into either an acute or blunt angle depending on the angle between the solid and adjacent cyst walls.

“Large comet-tail artifacts” are echogenic foci with V-shaped echoes that are greater than 1 mm deep. Macrocalcifications are coarse echogenic foci greater than 1 mm in diameter accompanied by acoustic shadowing. Microcalcifications are punctate echogenic foci less than or equal to 1mm in diameter with non-shadowing. Peripheral calcifications lie along all or part of a nodule's margin.

The solid component's echogenicity was classified as iso-echogenicity, hyperechogenicity, hypoechogenicity and marked (or very) hypoechogenicity by comparing the echogenicity of the solid component with thyroid parenchyma or strap muscles. The margins were further divided into well-circumscribed (smooth), ill-defined, lobulated, irregular, and extra-thyroid extension. The shape was assessed by the ratio of anteroposterior (A) and transverse (T) diameter (A/T≥1 or A/T<1) of the nodule. A regular, hypoechoic halo presented with a regular smooth profile corresponding to the pericapsular arrangement of nodule vascularity.

FNA was performed under US guidance, using a 23-gauge needle by an experienced endocrinologist. If a nodule was predominantly cystic, FNA for the solid portion was done after the fluid was aspirated. Afterward, the cytology was interpreted according to the Bethesda System for Reporting Thyroid Cytopathology (BSRTC).

### Statistical analysis

The statistical analysis was performed using the SPSS Statistics 20.0 for Windows (IBM SPSS statistics). Values are expressed as mean ± SD for continuous variables and as proportions for categorical variables (%). Groups were compared using independent-sample t-test, chi-square, and Fisher's exact test depending on the distribution. We performed binary logistic regression after univariable analysis to determine independent predictors for malignancy in PCTNs. Sensitivity, specificity, positive, and negative predictive values for malignancy and accuracy were calculated for US features. Statistical significance was set for P-value < 0.05.

## RESULTS

A total of 91 patients (68 females, 23 males) with 94 nodules were included in this study and ages ranged from 14 to 78 (mean age, 43.4 ± 13.1 years). There were solitary nodules in 88 patients and two nodules in three patients. The mean nodule size was 2.9 ± 1.2 cm (range, 1.1-8.6 cm).

The cytologic and histopathologic correlation is summarized in Table 1. Based on cytology, all the nodules were categorized as follows: nondiagnostic (cyst fluid only) (n=5), benign (n=29), atypia of undetermined significance/follicular lesion of undetermined significance (AUS/FLUS) (n=7), follicular neoplasm/suspicious for a follicular neoplasm (FN/SFN) (n=2), suspicious for malignancy (n=21), malignancy (n=30). The four categories nondiagnostic, AUS/FLUS, FN/SFN, and suspicious for malignancy, were considered indeterminate cytology. None of the nodules with nondiagnostic and benign cytology were malignant after thyroid surgery. The frequency of malignancy for AUS/FLUS, FN/SFN, suspicious for malignancy, and malignancy were 28.6%, 50.0%, 47.6%, and 96.7%, respectively.

**Table 1:** Cytological and histopathological results of all thyroid nodules

Cytology	Benign (n = 52)	Malignancy (n = 42)	Malignancy %
ND/UNS	5 (9.6%)	0 (0.0%)	0.0%
Benign	29 (55.7%)	0 (0.0%)	0.0%
AUS/FLUS	5 (9.6%)	2 (4.8%)	28.6%
FN/SFN	1 (1.9%)	1 (2.4%)	50.0%
Suspicious for malignancy	11 (21.2%)	10 (23.8%)	47.6%
Malignancy	1 (1.9%)	29 (69.0%)	96.7%

ND: nondiagnostic; UNS: unsatisfactory; AUS/FLUS: atypia of undetermined significance/follicular lesion of undetermined significance; FN/SFN: follicular neoplasm/suspicious for a follicular neoplasm.

US features and histopathologic results are summarized in Table 2. Univariable analysis was performed to determine sonographic features associated with a malignant nodule. The hypoechogenicity and microcalcification showed a statistically significant association with malignant nodules (P < 0.05). The prevalence of malignancy was significantly higher in the nodules with a solid portion greater than or equal to 50% (P = 0.001). An eccentric solid component with an acute angle showed a slight increase in malignant nodules (P = 0.048). However, neither overall analysis nor stratified analysis based on the percent of solid portion (≥50% or <50%, data not shown) showed a significant association of an eccentric configuration with malignancy. The size of the nodule, shape, margin, and halo presence were not significantly associated with a malignant nodule. Binary logistic regression analysis demonstrated that microcalcification and hypoechogenicity were significantly associated with malignancy. There was no significant association of age and sex with either benign or malignant nodules.

**Table 2:** US features of benign and malignant partially cystic thyroid nodules

US features		Benign (n = 52)	Malignancy (n = 42)	P-value	
				Uni-	Multi-
Mean size (cm)		3.18	2.62	0.181	0.275
Age (years)		47.02	38.93	0.909	0.080
Sex (female/male)		40/10	28/13	0.279	0.816
Composition					
	Solid portion ≥ 50%	30 (44.8%)	37 (55.2%)	**0.001**	0.081
	Solid portion < 50%	22 (81.5%)	5 (18.5%)		
Position of the solid portion					
	Eccentric	18 (62.1%)	11 (37.9%)	0.501	0.324
	Non-eccentric	34 (52.3%)	31 (47.7%)		
Eccentric configuration (n = 29)					
	With an acute angle	4 (36.4%)	7 (63.6%)	**0.048**	–
	With a blunt angle	14 (77.8%)	4 (22.2%)		
Shape					
	Taller than wide	0 (0.0%)	1 (100%)	0.447	1.000
	Wider than tall	52 (55.9%)	41 (44.1%)		
Margin					
	Smooth	46 (60.5%)	30 (39.5%)		
	III-defined	5 (38.5%)	8 (61.55)	0.084	0.445
	Lobulated or irregular	1 (20.0%)	4 (80.0%)		
Echogenicity					
	Hiper- or isoechoic	39 (68.4%)	18 (31.6%)	**0.002**	**0.034**
	Hypoechoic	13 (35.1%)	24 (64.9%)		
Containing calcification					
	None	41 (73.2%)	15 (26.8%)		
	Macro-	2 (40.0%)	3 (60.0%)		
	Peripheral (rim)	1 (100.0%)	0 (0.0%)	**< 0.001**	**0.027**
	Micro	8 (25.0%)	24 (75.0%)		
The presence of halo					
	Yes	5 (62.5%)	3 (37.5%)	0.728	0.830
	No	47 (54.7%)	39 (45.3%)		

Uni-: univariable analysis; Multi-: binary logistic regression analysis.

We also evaluated the diagnostic values of three sonographic features, which were significantly associated with malignant nodules in partially cystic thyroid nodules. Among them, microcalcification revealed the highest PPV (75.0%), accuracy (72.3%), but low sensitivity (57.1 %) as shown in Table 3. A combination of any two of the three sonographic features showed that microcalcification and hypoechogenicity exhibited the highest specificity (65.4 %), PPV (65.4 %), NPV (81.0 %), and accuracy (72.3%). When compared with the combination of all three features, there was an increased sensitivity (97.6%) and NPV (94.4%), but slightly decreased accuracy (61.7 %). Moreover, we evaluated these three features' diagnostic value in the nodules with indeterminate cytology (Table 4) and the results were like that of all nodules. The combination of the three features showed high sensitivity (92.3%) and NPV (83.3%). These results imply that there is no need for a repeat FNA for PCTNs with indeterminate cytology in the absence of these three features.

**Table 3:** Diagnostic efficacy of US features in partial cystic thyroid nodules

US features	Sensitivity	Specificity	PPV	NPV	Accuracy
a. Microcalcification	57.1%	84.6%	**75.0%**	71.0%	**72.3%**
b. Solid portion ≥ 50%	88.1%	42.3%	55.2%	81.5%	62.8%
c. Echogenicity	57.1%	75.0%	64.9%	68.4%	67.0%
a or b	92.9%	36.5%	54.2%	86.4%	61.7%
b or c	92.9%	38.5%	54.9%	87.0%	62.8%
a or c	81.0%	65.4%	65.4%	81.0%	72.3%
a or b or c	**97.6%**	32.7%	53.9%	**94.4%**	61.7%

**Table 4:** Diagnostic efficacy of US features in these nodules with indeterminate nodules

US features	Sensitivity	Specificity	PPV	NPV	Accuracy
a. Microcalcification	38.5%	81.8%	**55.5%**	69.2%	**65.7%**
b. Solid portion ≥ 50%	69.2%	27.3%	36.0%	60.0%	42.9%
c. Echogenicity	53.8%	59.1%	43.8%	68.4%	57.1%
a or b	84.6%	27.3%	40.7%	75.0%	48.6%
b or c	76.9%	22.7%	37.0%	62.5%	42.9%
a or c	76.9%	50.0%	47.6%	78.6%	60.0%
a or b or c	**92.3%**	22.7%	41.4%	**83.3%**	48.6%

## DISCUSSION

Partially cystic thyroid nodules are common findings in ultrasonographic examination (7,15). The findings from previous studies suggest the suspicious US features of mixed cystic solid thyroid nodules were different from that of solid nodules. The 2015 American Thyroid Association (ATA) guidelines have addressed the clinical management of partially cystic thyroid nodules based on a small number of studies (6). According to the 2015 ATA guidelines, PCTNs were recommended for FNA if greater than or equal to 1cm, or if the solid hypoechoic component included one or more features such as irregular margins, microcalcifications, a taller than wide shape, rim calcifications with small extrusive soft tissue components, and evidence of extrathyroidal extension (ETE). Most of these suspicious sonographic features were more common in solid malignant nodules and less frequent in partially cystic thyroid nodules. Our results revealed a marked increase in hypoechogenicity and microcalcification in the solid component of malignant nodules. Combining these two features showed 72.3% accuracy and 81.0% NPV for PCTNs. The Mayo clinics reported that nodules with the solid portion occupying less than 50 % of the volume occurred in only 2.5% of 360 consecutively surgically removed thyroid cancers (16). Our findings also showed that malignancy was less frequent in nodules with a solid portion of less than 50%. Also, using the combination of hypoechogenicity, microcalcification and composition showed an NPV of 94.4%. This indicates that the absence of these three features is a low risk for malignancy and only observation without an FNA is needed.

Eccentric configuration with an acute angle has been associated with malignancy in PCTNs (7,8,15,17). In this study, eccentric configuration with an acute angle rather than a blunt angle was slightly more frequent in the malignant nodules. In the 2015 ATA guidelines, PCTNs with an eccentric solid portion had an estimated 5-10% risk; they were categorized as having low suspicion if there were no microcalcification, irregular margin, and a taller than wide shape. In contrast, this sonographic feature was not considered in other ultrasonographic risk stratification systems including the 2016 Korean Society of Thyroid Radiology (18), the 2017 American College of Radiology (19), and the 2017 European Thyroid Association Guidelines (20). Hence, it was not independently predictive of malignancy in these nodules.

A taller than wide shape and lobulated/irregular margins were less frequently seen in PCTNs, although they were well-established for predicting malignancy in solid thyroid nodules. In concordance with previous studies, they were not significantly correlated with the malignancy of mixed cystic solid nodules. These sonographic features may have a limited role in differentiating malignant from benign mixed thyroid nodules.

It is also worth mentioning that microcalcification had the highest accuracy but the lowest sensitivity than the other two US features in PCTNs with indeterminate cytology. Combined with echogenicity and a solid portion greater than or equal to 50%, the sensitivity and NPV were increased to 92.3% and 83.3%, respectively. A repeat FNA might not be considered for nodules with indeterminate cytology if these three features in the solid component were absent. In summary, these suspicious sonographic features will help guide clinical decision-making when managing PCTNs with indeterminate cytology.

There were a few limitations of this study. First, this retrospective study did not include PCTNs without histopathologic results; second, there was a small sample size; and third, the FNA decision-making criteria were not all based on the 2015 ATA guidelines (some FNAs done were based on the patients' choice). Finally, most patients with PCTNs were diagnosed with conventional PTC and only one patient had a follicular variant of PTC.

In conclusion, this study demonstrated that microcalcification and hypoechogenicity were independently associated with malignancy in partially cystic thyroid nodules. The combination of microcalcification, hypoechogenicity, and a solid portion greater than or equal to 50% will be helpful to make clinical decisions in mixed cystic solid nodules.
